# Translationale Herausforderungen und klinisches Potenzial von künstlicher Intelligenz in der minimal-invasiven Chirurgie

**DOI:** 10.1007/s00104-025-02366-0

**Published:** 2025-08-26

**Authors:** Matthias Carstens, Micha Pfeiffer, Stefanie Speidel, Marius Distler, Jürgen Weitz, Fiona R. Kolbinger

**Affiliations:** 1 Klinik und Poliklinik für Viszeral‑, Thorax- und Gefäßchirurgie, Universitätsklinikum Carl Gustav Carus an der Technischen Universität Dresden, Dresden, Deutschland; 2 Abteilung für Translationale Chirurgische Onkologie, Nationales Centrum für Tumorerkrankungen (NCT/UCC) Dresden, Dresden, Deutschland; 3 Weldon School of Biomedical Engineering, Martin C. Jischke Hall of Biomedical Engineering, Purdue University, 206 S. Martin Jischke Drive, 47907-2032 West Lafayette, IN USA

**Keywords:** Deep Learning, Klinische Translation, Künstliche neuronale Netzwerke, Black Boxes, Regulatorische Rahmenbedingungen, Deep learning, Clinical translation, Artificial neuronal networks, Black boxes, Regulatory framework conditions

## Abstract

Künstliche Intelligenz (KI) bietet enormes Potenzial für die Chirurgie. Anwendungsfelder reichen von interdisziplinärer Therapiestratifizierung über die Unterstützung der Operationsplanung bis zur Entscheidungsunterstützung im Operationssaal, die im Fokus dieses Beitrags steht. Künstliche neuronale Netzwerke zur Analyse chirurgischer Videos können chirurgische Sicherheit, Effizienz und Planbarkeit verbessern. Voraussetzung dafür sind hochwertige, vielfältige (Meta‑)Daten, deren Annotation, Training und Validierung komplexe Anforderungen stellen. Trotz technischer Fortschritte scheitert die klinische Umsetzung bis dato oft an fehlender Datenstandardisierung, unzureichender Infrastruktur, regulatorischen Hürden und ethischen Unsicherheiten. Viele Modelle bleiben Black Boxes, was Akzeptanz und Vertrauen hemmt. Systeme müssen zudem robust, transparent und praktikabel in klinische Abläufe integrierbar sein. Um die klinische Translation von KI in der Chirurgie zu fördern, sind konsequente Datenerhebungsstrategien, datenschutzkonforme Lernverfahren, *Explainable AI* und *Human-in-the-loop*-Ansätze entscheidend. Auch regulatorische Rahmenbedingungen wie die EU Medical Device Regulation bzw. das Medizinprodukterecht-Durchführungsgesetz und der EU AI Act müssen KI-spezifisch für den medizinischen und insbesondere den interventionellen Bereich weiterentwickelt werden, um sichere, interdisziplinäre Assistenztechnologien im Operationssaal zu ermöglichen, die den chirurgischen Alltag sinnvoll ergänzen.

## Abstract (en)

Artificial intelligence (AI) holds great potential for minimally invasive surgery, with fields of application ranging from interdisciplinary treatment stratification through preoperative planning up to active decision support in the operating room, which are the focus of this article. Artificial neural networks for analysis of surgical video recordings could enhance surgical safety, efficiency and planning. High-quality, diverse (meta)data are essential for such AI applications but the annotation, training and validation present complex demands. Despite technological advances, the clinical implementation often fails due to a lack of data standardization, insufficient infrastructure, regulatory barriers and ethical uncertainties. Many models remain black boxes, which hinders acceptance and trust among medical professionals. In addition, AI systems need to be robust, transparent and practically integrable into clinical workflows. Stringent data collection strategies, privacy-preserving learning methods, explainable AI and human-in-the-loop approaches are critical to facilitate clinical translation. Regulatory framework conditions, such as the General Data Protection Regulation, the EU Medical Device Regulation and the EU AI Act, will require further legal refinements to address the specific needs of medical AI applications and interventions, to facilitate the safe adoption of interdisciplinary assistive technologies in the operating room that meaningfully support surgical practice.

Seit den 2010er-Jahren gewinnen künstliche Intelligenz (KI)-gestützte klinische Anwendungen zunehmend an Bedeutung und gelten als Wegbereiter für den Wandel medizinischer Praxis [21]. KI-Methoden finden in nahezu allen medizinischen Bereichen Anwendung, etwa bei der radiologischen Diagnostik [28], Transkription medizinischer Gespräche oder zur Entwicklung personalisierter Therapien [1]. Die minimal-invasive Chirurgie bietet durch das Vorliegen digital-visueller Daten operativer Prozeduren vielfältige Möglichkeiten für Computer-Vision-Technologien im Operationssaal. Dennoch sind solche Anwendungen bislang kaum in der klinischen Routine angekommen.

In der wissenschaftlichen Fachliteratur findet sich inzwischen eine Vielzahl an Arbeiten zur computergestützten Analyse und Interpretation visueller und kontextueller Informationen aus dem OP (Operations)-Umfeld. Es wurde bereits gezeigt, dass künstliche neuronale Netzwerke komplexe anatomische Strukturen präzise erkennen und visuell hervorheben können [15], etwa autonome Nerven bei kolorektalen Eingriffen [22], Adhäsionen bei Gastrektomien [17], relevante Dissektionsbereiche bei Rektumresektionen [14] oder sichere Präparationszonen bei laparoskopischen Cholezystektomien [18], um potenziell intraoperative Komplikationen zu vermeiden. Der Einsatz KI-basierter Anwendungen verspricht, die chirurgische Sicherheit zu erhöhen, das Patientenoutcome zu verbessern, Abläufe zu optimieren und Kosten zu senken [3, 19].

## Training und Informationsverarbeitung mit Methoden künstlicher Intelligenz

**Abb. 1:**
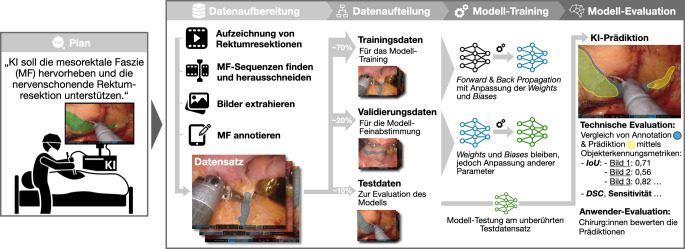
Übersicht über die Entwicklung einer KI-Anwendung am Beispiel der Erkennung der mesorektalen Faszie bei minimal-invasiven Rektumresektionen.* IoU* Intersection-over-Union, *DSC* Dice Similarity Coefficient

Moderne KI-Methoden verarbeiten entweder einen Datentyp (unimodale Modelle) oder mehrere unterschiedliche Datentypen (multimodale Modelle). Je nach Anwendungszweck kommen für intraoperative KI-Modelle verschiedene Bild- und Videodaten (z. B. Laparoskopie, Radiologie), Text- oder Audiodaten infrage [19]. Hochwertige Trainingsdatensätze sind klinisch repräsentativ, enthalten Patientenmetadaten, stammen aus verschiedenen Institutionen und wurden standardisiert erhoben, geprüft und verarbeitet. Bei visuell interpretierenden KI-Modellen umfasst die Datenverarbeitung häufig (Experten‑)Annotationen, etwa zur Sichtbarkeit und Lokalisation chirurgischer Instrumente, anatomischer Strukturen oder Handlungsschritte (Abb. 1). Annotationen können manuell, halbautomatisch oder KI-gestützt erfolgen, z. B. durch Markieren spezifischer Bildinhalte oder binäre Klassifikationen wie „Instrument sichtbar: ja/nein“. Ergänzend können Metadaten wie postoperative Komplikationen oder histologische Befunde ins Training einfließen oder als Prädiktionsziele dienen.

Neuronale Netzwerke werden traditionell iterativ trainiert, wobei Modellparameter (sog. *weights* und *biases*) der künstlichen Neurone schrittweise angepasst werden. In der Forward Propagation werden Eingabedaten durchs Netzwerk geleitet, um Vorhersagen zu erzeugen. In der anschließenden Back Propagation wird der Fehler berechnet durch den Vergleich mit den manuell erstellten Annotationen. Daraus ergibt sich eine Anpassung der Modellparameter zur Verbesserung künftiger Vorhersagen (Abb. 1; [16]).

Vor dem Training wird der Datensatz in Trainings‑, Validierungs- und Testdaten geteilt. Das Trainingsset dient der Parametrisierung, das Validierungsset der Feinjustierung, das unabhängige Testset der finalen Leistungsbewertung des Modells. Zur Sicherung der Unabhängigkeit des Testsets erfolgt diese Aufteilung der verfügbaren Daten in aller Regel auf Patientenebene oder Institutsebene, d. h., alle Daten einer Person bzw. eines Zentrums befinden sich entweder im Trainings-, im Validierungs- oder im Testset. Je nach Zielstellung werden Aufgaben wie Klassifikation (z. B. „Risikoorgan vorhanden: Ja/Nein“), Segmentierung (z. B. räumliche Abgrenzung von Strukturen im Bild) oder Regression (z. B. Tumorgrößenschätzung) bearbeitet. Die Trainingsart, überwachtes, teil- oder selbstüberwachtes Lernen, beeinflusst sowohl den Annotationsaufwand als auch die Modellqualität. Jede Methode bringt spezifische Vor- und Nachteile mit sich (Abb. 1; [16]).

Die Datenqualität für das Training von KI-Modellen ist für dessen klinische Nutzbarkeit entscheidend

Zur Leistungsbeschreibung werden meist technische Metriken wie die *Intersection-over-Union* (IoU) oder der *Dice Similarity Coefficient* (DSC) verwendet. Sie quantifizieren, wie stark die KI-Vorhersage mit der sog. Grundwahrheit (*ground truth*) übereinstimmt, also dem gemessenen oder als ideal definierten Referenzwert (Abb. 1; [20]). Für die klinische Relevanz ist zudem entscheidend, wie robust das Modell unter Realbedingungen arbeitet und wie stark seine Leistung etwa mit Patientenmerkmalen, Bildqualität oder Klinikstandort variiert. Auch die Bestimmung von Modellunsicherheiten gewinnt an Bedeutung, um Vertrauen in die KI-Vorhersagen einschätzen zu können [6].

## Klinisches Potenzial von künstlicher Intelligenz in der (minimal-invasiven) Chirurgie

**Abb. 2:**
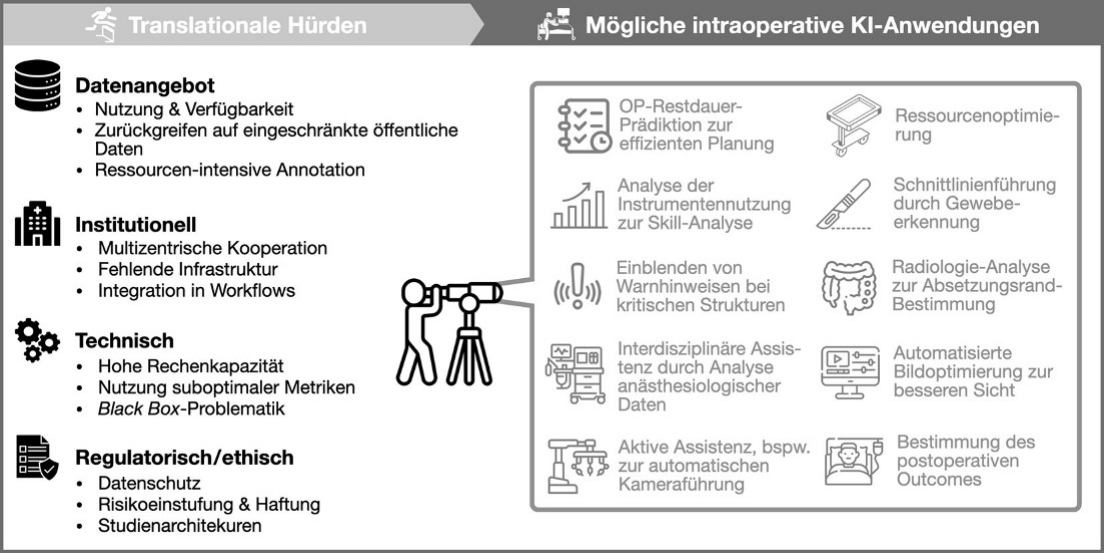
Herausforderungen für die klinische Translation intraoperativer KI-Systeme sowie mögliche Anwendungsbeispiele

In der minimal-invasiven Chirurgie sind auf Basis klinischer Routinedaten zahlreiche KI-Anwendungen denkbar, die das Potenzial haben, die Praxis entlang des chirurgischen Behandlungspfades grundlegend zu verändern (Abb. 2):

*Präoperative KI-Systeme* könnten die OP-Planung optimieren. So ließen sich anhand radiologischer Daten Resektionsränder bereits vor dem Eingriff anhand von Tumorausbreitung und Metastasierung bestimmen. Zudem ermöglichen patientenspezifische 3D-Darstellungen von Gefäß- und Organstrukturen exaktere Schnitte und schonendere Absetzungsränder [27]. Ergänzend schätzen KI-Modelle präoperativ individuelle Risiken postoperativer Komplikationen anhand von Patientendaten ab [13].

KI-Anwendungen können die Praxis entlang des chirurgischen Behandlungspfades grundlegend verändern

Die Forschung zur KI-gestützten Interpretation von OP-Videos und *intraoperativer Bildverarbeitung* fokussiert bislang meist auf deskriptive Analysen visueller Informationen zur Instrumenten- oder Risikostrukturerkennung, meist noch ohne klinische Anwendung [14]. Perspektivisch könnten KI-Systeme chirurgische Fähigkeiten interpretieren, Warnhinweise bei Gefährdung ausgeben und die Bildqualität durch Rauschreduktion oder Farbkorrektur verbessern [2]. Modelle auf OP-Video-Basis könnten zudem Force-Feedback in der robotischen Chirurgie ermöglichen, um fehlendes taktiles Empfinden zu kompensieren, etwa durch das Erlernen optischer Gewebeveränderungen bei Druckeinwirkung [7].

Durch Erkennung chirurgischer Phasen sind *prozessbezogene Assistenzsysteme* denkbar, etwa zur Vorhersage benötigter Instrumente [30], was OP-Planung, Ressourcenverteilung und Kommunikation im Saal verbessern könnte. Auch die voraussichtliche OP-Restdauer ließe sich prognostizieren, um Folgeeingriffe oder Personalbedarf effizient zu planen [25].

*Aktive intraoperative Assistenzsysteme* im Sinne einer *embodied AI* befinden sich noch im Frühstadium. Sie kombinieren KI-gesteuerte Bildanalyse mit Aktoren, um bei Bedarf direkt einzugreifen, z. B. durch automatisierte Kameraführung oder verbesserte Exposition relevanter Strukturen [11]. OP-Roboter bieten dafür eine ideale Basis, da ihre präzise Steuerung mit KI-Software verknüpft werden kann.

Multimodale KI-Systeme integrieren verschiedene Datenquellen zur Entscheidungsunterstützung. Sprach-KI könnte während Operationen Hinweise geben [9] oder Informationen aus Patientenhistorie, Medikamentenplänen und OP-Prozessen nutzen, um kontextbezogene Empfehlungen auszusprechen oder unter Berücksichtigung aller am Ende der Operation vorliegenden Daten *postoperative* *Risiken* einzuschätzen [24].

## Translationale Herausforderungen

Trotz des Potenzials intraoperativer KI in der minimal-invasiven Chirurgie bleibt der Einfluss auf die Patientenversorgung gering. Von über 1000 von der U.S. Food and Drug Administration (FDA) zugelassenen KI-gestützten Medizinprodukten sind nur wenige für den intraoperativen Einsatz bestimmt, darunter das Triton System (Gauss Surgical, Inc., Menlo Park, Kalifornien, USA) zur Echtzeitblutverlustmessung, das NvisionVLE Imaging System (NinePoint Medical, Inc., Austin, Texas, USA) zur Segmentierung bei gastroenterologischen Eingriffen und Proprio PARADIGM (Pass Vision, LLC, Seattle, Washington, USA) zur strahlungsfreien 3D-Navigation bei Wirbelsäulenoperationen. In Japan wurde EUREKA (Anaut, Inc., Tokio, Japan) zur Erkennung von Adhäsionen und empfindlichem Gewebe zugelassen, jedoch nur zu Lehrzwecken.

Dass bislang nur wenige Systeme in der klinischen Anwendung angekommen sind, liegt v. a. an begrenztem Datenzugang, fehlender standortübergreifender Zusammenarbeit, technischen Hürden sowie regulatorischen und ethischen Herausforderungen (Abb. 2). Die Entwicklung klinisch relevanter KI erfordert über Jahre hinweg eine enge Kooperation zwischen Medizin, Technik und Regulatorik. Entsprechende Bedingungen bestehen international nur an wenigen Standorten.

Trotz des Potenzials der laparoskopischen Bild- und Videodaten werden OP-Videos in den meisten Kliniken nicht standardmäßig aufgezeichnet, etwa aus Speicherplatzgründen oder aufgrund technischer und personeller Engpässe [29]. Hinzu kommen Herausforderungen wie Bewegungsartefakte, variable Bildqualität oder heterogene OP-Ansätze. Öffentliche Datensätze sind zwar zunehmend verfügbar, meist jedoch monozentrisch, klein, wenig variabel und ohne relevante Metadaten wie Komplikationen veröffentlicht [5]. Oft beschränken sie sich auf Cholezystektomien mit Instrumentenannotationen, was die klinische Realitätsnähe einschränkt [12]. Sie ermöglichen zwar kontrollierte Machbarkeitsstudien, führen aber durch wiederholte Nutzung zu einer verzerrten Forschungsumgebung, die die Vielfalt chirurgischer Eingriffe nicht abbildet und Zweifel an der klinischen Validität aufwirft [14].

Zur Maximierung des klinischen Nutzens müssen Daten standort- und patientengruppenübergreifend sowie variabel erhoben werden. Zukünftig sollten Trainingsdaten mit Informationen zu Outcome und technischen Parametern ergänzt werden. Internationale Initiativen wie die CVS Challenge (https://www.cvschallenge.org) sammeln bereits OP-Videos zur objektiven Bewertung von KI-Modellen bei der Erkennung des Critical View of Safety. Datenschutzkonforme Ansätze wie dezentrales oder teilüberwachtes Lernen könnten die Entwicklung beschleunigen, den Annotationsaufwand senken und mehr Kliniken die Teilnahme am Entwicklungsprozess ermöglichen.

Technische Herausforderungen erschweren zusätzlich die Integration in den OP-Saal. Kleinste Modellfehler können in der Chirurgie schwerwiegende Folgen für die Patientensicherheit haben. Viele Krankenhäuser verfügen weder über passende Ausstattung noch über Infrastruktur zur Datenverarbeitung oder -weitergabe. Die Integration in bestehende Abläufe ist komplex, da Auswertungen in Echtzeit erfolgen müssen, was hohe Rechenleistung erfordert [19].

Zudem ist die Abschätzung der klinischen Modellleistung schwierig. Technische Metriken wie IoU oder DSC liefern kaum Aussagen zum tatsächlichen Nutzen oder dem Einfluss auf klinische Endpunkte [19, 26]. Klinisch relevante Aspekte wie Sicherheit, Akzeptanz und Umsetzbarkeit sowie Einfluss chirurgischer KI-Modelle auf objektivierbare und relevante Endpunkte müssen in (prospektiven) Nutzerstudien untersucht werden. Die Black-Box-Eigenschaft vieler Modelle erschwert das Vertrauen des medizinischen Personals, da nicht nachvollziehbar ist, wie KI-Entscheidungen zustande kommen. *Explainable AI* kann Entscheidungsprozesse nachvollziehbarer machen; Human-in-the-loop-Ansätze integrieren Fachwissen bereits im Training, verringern Verzerrungen und sichern ethische Standards [4, 23].

Die Black-Box-Eigenschaft vieler Modelle erschwert das Vertrauen des medizinischen Personals

Auch regulatorische und ethische Fragen sind zentrale Herausforderungen. Der Umgang mit Patientendaten wirft Fragen zu Sicherheit, Eigentum und ethischer Nutzung auf. Zwar existieren gesetzliche Rahmenwerke wie die Datenschutz-Grundverordnung (DSGVO), das Medizinprodukterecht-Durchführungsgesetz (MPDG) oder der Health Insurance Portability and Accountability Act (HIPAA), sie bleiben jedoch vage hinsichtlich dynamischer KI-Systeme mit sich ändernden Outputs. Der EU AI Act (2023) führt allgemeine Risikostufen für KI ein, berücksichtigt aber medizinische Anwendungen nicht explizit. Die FDA klassifiziert KI als *Software-as-a-Medical-Device (SaMD)*, fordert eine klare Zweckdefinition und evidenzbasierte Nutzenbewertung, eine Herausforderung angesichts der Black-Box-Problematik und variabler Modellleistung bei seltenen Fällen, da die KI-Leistungsfähigkeit stark von den verwendeten Trainingsdaten abhängt [10]. Haftungsfragen sind international uneinheitlich geregelt, zudem fehlen klare Leitlinien für den klinischen Translationsprozess und das Design prospektiver Studien KI-basierter Medizinprodukte [8].

Die klinische Translation von KI-Modellen für die Chirurgie ist mit spezifischen Herausforderungen verknüpft. Um den Einsatz von KI in der chirurgischen Routine zu bahnen und prospektive klinische Studien zu ermöglichen, die den Einfluss von KI auf die Versorgungsqualität erfassen, muss ein enger Austausch mit Chirurginnen und Chirurgen sowie medizinischem Personal gewährleistet sein. Nur wenn ihre Erfahrungen und Bedarfe von Beginn an einfließen, kann das volle Potenzial von KI im chirurgischen Alltag zur Entfaltung kommen.

## Fazit für die Praxis

- Für klinisch relevante chirurgische KI-Modelle werden diverse, multizentrische Datensätze benötigt, die mit relevanten Metadaten verknüpft und datenschutzkonform ausgetauscht werden.
- Der Annotationsaufwand sollte reduziert werden: Teilüberwachtes Lernen und automatisierte Annotation können die KI-Entwicklung erleichtern und die Datenbasis erweitern.
- Vertrauen muss geschaffen werden: *Explainable AI* und *Human-in-the-loop*-Ansätze helfen, die Black Box KI zu öffnen, klinisches Wissen zu integrieren und transparente, ethisch fundierte KI-Modelle zu entwickeln.
- Regulatorische Rahmenbedingungen müssen angepasst werden: Aufbauend auf existierenden Rahmenbedingungen wie dem EU AI Act und FDA-Guidelines ist eine klarere Definition der translationalen Schritte von präklinischer Entwicklung zur klinischen Anwendung notwendig.
